# Dr. Lydston Wins: The Machine Is “Busted”

**Published:** 1913-11

**Authors:** 


### EDITORIAL DEPARTMENT.

**DR. F. E. DANIEL, Editor** **DR. R. H. L. BIBB, Associate Editor**

DEPARTMENTS { EUGENICS: Drs. M. Duggan and T. Y. Hull.

WOMAN'S DEPARTMENT: Mrs. F. E. Daniel.

DR. LYDSTON WINS: THE MACHINE IS "BUSTED."—A modern St. George has arisen to slay the dragon. In the case of G. Frank Lydston vs. John C. W. Wayman, State's Attorney, Cook county, Ill., in mandamus to compel service of quo warranto writs on the trustees of the A. M. A. the appellate court handed down the opinion (Oct. 9, 1913) that the A. M. A. is holding meetings contrary to law, that its elections and its delegate system are illegal and that each and every member is entitled to a direct vote.

Unless the Supreme Court comes to the rescue this means that, for nearly fifteen years, the A. M. A. has been conducting its business illegally. This will necessitate a complete reorganization of the A. M. A. and the return of the association to its members, from whom the oligarchy stole it nearly fifteen years ago at St. Paul. The officers of the A. M. A. have for nearly four years been fighting against a decision of the question of the legality of the operations of this association. Why? The points decided by the court in Dr. Lydston's favor, were embodied in the famous "reform" resolutions which the "gang" fought so hard to prevent him from presenting to the Illinois State Medical Society several years ago. It seems that Dr. Lydston succeeded in getting in the courts an audience which he could not get elsewhere.

In a note accompanying the above, Dr. Lydston says:

"The foregoing is correct. I hope to return the A. M. A. to the membership in the near future."

I reproduce the two following editorials from the Chicago papers which will give my readers all the information on the subject:

"A decision which, if sustained, will bring about drastic reforms in the business procedure of every corporation incorporated under the laws of the State of Illinois, has been handed down by the appellate court of Cook county in the case of Dr. G. Frank Lydston against the officers of the American Medical Association.

"In brief, the decision means that in the future all elections of officers of corporations incorporated under the laws of Illinois must be held in the State and that every stockholder or member is entitled to a direct vote, either in person or by proxy, at these elections.

"Circuit Court Reversed: The finding of the appellate court is a complete reversal of the decision of the circuit court, in which Dr. Lydston started quo warranto proceedings three years ago to force the late John E. W. Wayman, then State's Attorney, to serve ouster writs on the trustees and officers of the American Medical Association.

"Dr. Lydston's contentions of illegalities, which are now upheld by the appellate court, for the removal of the officers and trustees of the A. M. A. are:

"1. That the elections of the A. M. A. are illegal, being held outside of the State of Illinois, in which the association was incorporated.

"2. That the majority initial vote in the association is cast by non-members.

"3. That the delegate system of the A. M. A. robs the individual members of their right to vote as members of the association.

"4. That every member of the association is entitled to a direct vote in person or by proxy.

" 'The decision if upheld means primarily that the American Medical Association will be restored to the members of the American Medical Association,' said Dr. Lydston last night. 'The association was filched from the members by an oligarchy nearly fifteen years ago, and since then the rank and file has had nothing to say in the conduct of its affairs.'

"Sees Large 'Trust.' 'Through operations which the appellate court holds illegal the men who control the association have been enabled to build up what is to all intents and purposes a trust monopoly. This statement, a rather broad one, is borne out by a bill recently introduced in Congress by Congressman Riley of Connecticut.

" 'Congressman Riley's bill provides for the establishment of a United States Medical Examining and Licensing Board composed entirely of medical officers of the army, navy and marine hospital corps. The requirements for the examination as submitted by the proposed law, are to be prescribed by the American Medical Association, and only such medical diplomas are to be recognized as are issued by colleges approved by the American Medical Association.' "—*Examiner,* October 25, 1913.

"Complete reorganization of the American Medical Association, a national organization chartered under the law of Illinois, with a membership of about 40,000 physicians, may result from a decision of the Illinois appellate court which became public yesterday. Dr.

G. Frank Lydston, who for three and a half years has waged a verbal and legal battle against certain officials of the organization and its manner of operation, was the acting factor in the suit which was decided October 7.

"The warfare between Dr. Lydston and the association began when the physician attempted to prevent Dr. George H. Simmons, then secretary, from holding three offices at once. In discussing the verdict of the court last night, Dr. Lydston outlined the effects of the decision and said:

" 'I am hoping to present the association upon a platter, in the near future, to the membership from which it was stolen almost fifteen years ago. Should there be a reorganization, for which I sincerely hope, I shall fight for the referendum, legal elections, a membership ballot and nominations by petition.'

"Explains Legal Action. 'The legal action which I took was a proceeding in mandamus to compel the late John E. W. Wayman, then State's Attorney, to serve quo warranto writs on the trustees of the association. Mr. Wayman refused to serve them, as did the Attorney General. The A. M. A., through its attorneys, then fought the mandamus proceedings for the State's Attorney, and the circuit court decided against me.

" 'The appellate court, however, has reversed this decision and given a sweeping opinion in the case in favor of my contentions, which were, first, that the membership of the association at large had no vote, and consequently no voice in the management of the association. This robbed 35,000 men of their rights.

" 'Second, the meeting and business transactions outside of the State of Illinois (the State of incorporation) were illegal, and therefore that the association had been operating illegally. Third, that the A. M. A. had no legal power to vest in the election of its trustees in delegates elected by its alleged constituent bodies. If this decision is upheld by the Supreme Court, to which I suppose the officers of the association will appeal it, the entire present organization of the A. M. A. must either dissolve or reorganize from the bottom up.'

"Majority of Non-Voters. 'Fourth, the grounds of my contentions were that the majority initial vote in the association was that of non-members. If the Supreme Court should sustain the appellate court's decision I shall be perfectly satisfied, for all I ever asked was that the officers of the association prove once and for all their operations were legal.

" 'My object has been to take the association out of the hands of an oligarchy and restore it to the members to whom it rightfully belongs.

" 'During all of the legal fight the A. M. A. has admitted the facts but denied that the legal objections were well founded, hence the mandamus proceeding carries with it the entire legal merits of the case.' "—*Inter-Ocean,* October 25, 1913.

